# Medical and Philosophical Intelligence

**Published:** 1814-06

**Authors:** 


### MEDICAL AND PHILOSOPHICAL INTELLIGENCE.

AT a general meeting of Apothecaries, &c. held at the Crown and Anchor Tavern, Strand, on Thursday, May 12th, 1814, Mr. BURROWS in the Chair, the following Report was brought up

 from from the General Committee, which it was moved and seconded should be now read.

*Fourth Report of the General Committee of Associated Apothecaries of England and Wales.*

The General Committee of associated Apothecaries of England and Wales, agreeably to the letter and spirit of the instructions of the General Meeting, held at this house November 20th, 1812, have, from time to time, published their proceedings in the form of Reports.

The second of these, dated September 4th, 1813, takes a full review of the causes which led to the withdrawing of the former bill; the principles which would guide the Committee in forming a new one; and the means best calculated to insure its success. To this is annexed a copious appendix, containing the correspondence of the Committee with the corporate medical bodies, and copies of the memorials addressed to induce them to assist with their council in arranging a new bill. This Report concluded with the Resolutions of the 4th of September, 1813.*

As the matter of the third Report, dated February 22d ultimo, containing the Report of the College of Physicians, is highly important, strictly relevant to the business of this day, and expedient to be fully remembered, the Committee beg to invite the particular attention of the meeting to its contents.† The Committee, during their deliberations and arduous exertions of eighteen months, have never for one moment swerved from the pursuit of the great object for which they conceived themselves appointed—the procuring of a legislative enactment that would, by its gradual operation, secure for the service of the public properly educated practitioners, and for their branch of the profession that respectability and consideration to which its usefulness to society is so justly entitled. Actuated by this wish only, the Committee passed the Resolution of November the 19th, 1813; that "they determined *not* to apply for the formation of a fourth medical body, for the purpose of examinations, if the powers of the present chartered bodies can be so extended as to accomplish the objects which the resolutions of the 4th of September, 1813, embrace."

Consequently, when the Report of the College of Physicians was received, they transmitted it to the Society of Apothecaries; at the same time inviting them to a conference; and a deputation, consisting of the Chairman, Mr. Field, Mr. Thomson, Mr. Upton, and Mr. Parkinson, were nominated for that purpose. On the part of the deputation certain questions were asked; to some of which, as the Committee of the Society were not previously acquainted with their import, they declined giving definitive answers; and it was therefore agreed they should be subsequently reduced to writing, and sent to the Hall. On the part of the Society, it was stated, that they, in conformity with the Report of the College of Physicians,

---

* The second Report, and these Resolutions, were inserted in the Medical Journals of October last. They were also printed and published separately, and copies were sent to all the district Committees.

† See the London Medical Journals for March last.

Physicians, would bring in a bill, at their own expence; and that it should be drawn up on the basis of the Resolutions of the Committee of Associated Apothecaries, of September the 4th, *as far as regarded the practice of the Apothecary;* and that they could see no objection to all persons, applying for certificates to practise, being examined in the science and practice of medicine, pharmacy, chemistry, materia medica, and medical botany.

No formal answer was returned to the questions which were sent; but the Chairman received a private and satisfactory assurance, that the Society were rapidly proceeding to complete the draft of a bill; a copy of which should be sent to him as soon as it was prepared.

When the Committee found that the subject was seriously undertaken by two of the medical bodies, whom they had importuned to unite in an application to Parliament, they suspended their proceedings for introducing a bill, till they learnt the result of the labours of the Society; and that the measure might be rendered as efficient as possible, the party occupied with its details were furnished with all the information in possession of the Committee.

On the 21st of last month, the draft of the bill being received from the Society of Apothecaries, it was maturely discussed in the Committee, and several material alterations and amendments were suggested; which the Committee feel it an act of justice to the College of Physicians, and to the Society of Apothecaries, to acknowledge, were immediately adopted, and are now embodied in the bill.

As the representatives of the great body of general practitioners, and who, from a most extensive correspondence and communication, may presume that they are well informed, both with the nature of the evils that influence the prosperity of our profession, and with the remedies that will probably correct them; as men who have long reflected on, and duly weighed all the difficulties of our situation, and the means most likely to obviate them; the Committee do agree and determine, that the bill now offered to your consideration, though not containing every thing that might be desired, is, upon the whole, ONE that will place the Apothecary in his proper rank in society, and eventually produce advantages more commensurate with the value of his services.

Impressed with this conviction, the Committee take leave to recommend "The BILL for enlarging the CHARTER of the SOCIETY of APOTHECARIES, &c. and for better regulating the PRACTICE of APOTHECARIES throughout ENGLAND and WALES," to the approbation of this meeting, and of the profession.

*May* 11*th*, 1814. G. M. BURROWS, Chairman.

The following Resolutions were passed:—

"Resolved, That this meeting do agree with the Committee in approving the basis and spirit of the bill now submitted to it, and which is about to be introduced to Parliament by the Society of Apothecaries, with the approbation of the College of Physicians; and do recommend to the Committee to watch the progress of it in Parliament, and to take such measures from time to time as they may think best adapted to promote the interests of the profession, and the welfare of the public."

 "Resolved,

"Resolved, That the best thanks of this meeting be presented to the Right Hon. GEORGE ROSE, for his great attention to the interests of the general practitioner in medicine; and that he be requested to continue his kind services in Parliament for the passing of legislative measures tending to the improvement of the different branches of the medical profession, and to the welfare of the public."

"Resolved, That the thanks of this meeting be given to the Chairman, for his attention to the business of the meeting, and for his impartial conduct in the chair."

(Signed) G. M. BURROWS, Chairman.
*May* 12*th*, 1814. W. T. WARD, Secretary.

[One of the clauses introduced by the learned College of Physicians in this bill, makes it imperative on the apothecary to prepare any and every prescription of the regularly-licensed physician.]

---

A strongly marked case of Elephantiasis of Aretæus, as described by Dr. ADAMS, is now in St. Bartholomew's Hospital, under the care of Sir James Earle. The subject is approaching the age of puberty, but the progress towards the changes which occur at that period has ceased altogether, and the testicles are scarcely perceptible. Though under the age of fifteen, his countenance exhibits the appearance of old age, from the deep rugæ which are so peculiarly characteristic of this disease.

---

A memoir on the use of Charcoal has been read to the Medico-Chirurgical Society, in which it is said to possess the properties of Cinchona in the cure of intermittents and some other complaints. We shall be happy to hear from our correspondents a confirmation of the fact: but we are sorry to add, that in a case of ague, in which it was tried by Dr. Marcet at Guy's Hospital, it was not attended with success, and the disease afterwards readily gave way to bark. We mention this on the authority of some members present at the meeting.

---

*Hydrophobia.*—The following melancholy case of this dreadful malady lately occurred in Newcastle:—James Sharp, glassman, son of Alex. Sharp, of Queen-street, complained of being unwell, on Wednesday, Dec. 8, in the morning, after returning from his work at the Northumberland glass-house; he said he had been vomiting throughout the previous night while at work. On Thursday he was much worse, when an emetic was procured for him, but he could not bear the sight of it when put into a liquid. On Friday, a medical man examined the youth, and trying the effect the sight of water produced on him, gave it as his opinion that it was a case of hydrophobia. Inquiry was then made whether he had ever been bitten by a dog, but the parents were ignorant of such a circumstance; the young man was then asked, when he said a puppy of his had bit his thumb three weeks ago last Sunday, and that the dog died soon after. A powder was now given him, which he swallowed with closed eyes and the greatest agitation, not being able to bear the sight of the water in which it was mixed. The case arrested the attention of several of the faculty; in the afternoon of Friday he was bled in both arms, and in the temple. From that time he continued excessively ill, till about half past three o'clock on Saturday morning, having only about about ten minutes respite between each paroxysm. A few minutes before expiring, he expressed a wish for a drink of *warm* water—about two teacupsful were given him, when he appeared something easier. Shortly after, he had a desire to make water; he was taken up for that purpose, but no sooner did his feet touch the ground, than he threw himself back in his father's and uncle's arms, and expired without a groan.

---

The scarcity of Leeches is such, that where topical bleeding is necessary, the patient suffers in consequence, which induces a correspondent to suggest, that the operation might undoubtedly be performed by means of a powerful syringe, which is the principle the leech acts upon, and a very acute perforator, and what the leech has, a protruding lip, which keeps the external air from the puncture; but any one that would wish to make the experiment, should study the anatomy of the leech.

---

Dr. Adams's pamphlet on Hereditary Diseases is published, with his Remarks on Dr. Bateman's Synopsis.

---

Some important observations have been presented to the French Institution on the anatomy of Insects, particularly respecting the use of the great vessel which almost all the class have along the back, and which is subject to motions of dilatation and contraction similar to those of the heart and the arteries. Malphigi and Swammerdam gave it the name of heart: but it appears, from the observations of Lyonnet and many others, that no branches issue from it; and Cuvier seems to have proved, by many experiments, that insects have no sort of circulation. M. Marcel de Serres has examined this subject anew, and satisfied himself, by innumerable experiments made on the largest insects of the South of France, and aided by the most delicate anatomical instruments, that the dorsal vessel has no ramification whatever; that there is not in the body any other contractile vessel, nor in general any system of blood-vessels.

If the dorsal vessel be taken away from the insects which possess it, they will still live many hours; while the scorpion and the spider, who have really a heart, perish immediately when that is destroyed. The contractions of the dorsal vessel are principally owing to the muscles of the back, which are placed along its sides: but the trachea and the nerves have a sensible effect upon it. The fluid which it contains appeared generally to be of a colour analogous to that greasy matter with which some other parts of the bodies of insects are always filled; and possesses little liquidity, particularly among the voracious larvæ. The diameter of the vessel has been found nearly cylindrical in those larva where the greasy matter has been most equally distributed; and the inequalities of its different parts were proportioned to those of the corresponding parts of the grease of the body. The nerves and the trachea abound more in the dorsal vessels of the larvæ than in those of perfect insects; and contractions in the former are stronger but less frequent. From these and some other facts, the author considers himself justified in concluding, that the function of the dorsal vessel is to produce the greasy matter; and, in order to perform this function, it absorbs a part of the nutritive liquor, which is diffused in the cavity of the body through the partitions of the intestine; and which causes it afterwards to transude through the meshes of the cellular tissue, where the grease receives its definitive elaboration.

---

M. Jourdan, Surgeon of the Imperial Guard, in conjunction with M. Alyon, has published, in Paris, a Translation of Professor Hecker's work on Gonorrhœa, with a preface and notes, which display considerable research.

In the historical part, it is proved on the authority of sacred history, the classic authors, and the early medical writers, that excretions of fluid from the urethra, mucous and puriform, had been noticed; but, that no distinct account of a disease known by the name of Gonorrhœa, is to be found, until after the appearance of the venereal disease in Europe.

The author enumerates fifteen species of Gonorrhœa, which may be ranged under four general heads.

1. Gonorrhœa, commonly so called, (Blennorhagia urethralis of Swediaur,) excited by the stimulus of a specific contagion.

2. A flow of puriform mucus from local irritation of the urethra, not contagious.

3. An occasional sympathetic action of the mucous glands of the urethra in scrofulous, gouty, and other subjects.

4. An increased secretion of mucus, or an escape of the seminal fluid, caused by an atonic state of the parts.

A very weak solution of caustic alkali, one grain to each ounce of water, which may be further diluted, is recommended as the best preventive of Gonorrhœa; or lime-water, with half a grain of oxymuriate of mercury to each ounce. One of these lotions is to be used immediately after coition, and employed frequently for several days.

The general management of the complaint consists in checking the inflammation at its commencement; but instead of injections, the use of sedative soluble bougies is recommended. These are prepared by combining the substances intended to be employed with a gum of easy solution in the mucus of the urethra. The basis of the bougie is a cotton, or worsted thread, about the length of a finger, upon which the composition is spread so as to form a soft and uniform covering.

"Dissolve four grains of caustic potass (kali purum) in two ounces of distilled water, and add a sufficient quantity of gum arabic (powdered) to make the solution viscid. Dip the pieces of cotton into the composition, and suspend them separately by pins, taking care that they retain a proper form; then dip them again when dry, and continue this process until they are of a convenient size to pass into the urethra."

Bougies may also be made by dissolving four grains of oxymuriate of mercury in two ounces of distilled water, adding a sufficient quantity of gum arabic to give them a proper consistence. A drachm of the aqueous extract of opium may be added to the solution of potass, or to the solution of mercury.

Bougies can also be made by the addition of opium to the plain mucilage; and these are found to be particularly useful where the urethra is much inflamed, or in a state of high irritation. Alum, acetate acetate of lead, and other astringent substances, can be equally blended and formed into bougies, and thus become beneficial in an atonic or weak state of the urethra. Extract of hyosciamus, or of belladona, either alone, or combined with the oxymuriate of mercury, deserve attention, in cases of severe pain, spasm, and obstinate inflammation of the canal.

Dr. Hecker considers that a double object is to be fulfilled by these bougies: the first, to allay acute pain; the second, to excite in the urethra, inflamed by a morbific contagious principle, an irritation which shall overcome its action, and remove the inflammation dependent on local debility. Following this theory, he recommends the bougies to be employed only before the inflammation is fully formed, or whilst it is moderate. They are to be moistened with saliva, or milk, gently passed beyond the seat of the disease, which is considered to be chiefly about the *fossa navicularis*, and permitted to remain in the urethra half an hour, or longer, until the coating is totally dissolved, when the thread is to be withdrawn, and another introduced, either immediately or after a short interval *(au bout de quelque temps)*. If the pain excited be slight and supportable, the instrument is to be retained, as this sensation soon subsides; but, if severe, it is to be withdrawn, being considered a proof that the bougie is either too large, or its ingredients are too stimulant. The patient should remain on the bed or sofa during the time of using these instruments: if at night they are to be secured by a tape in the same manner as common bougies.

During the first period of Gonorrhœa, and before the inflammation is considerable, bougies made of potass and opium are said to be the most proper. If they be employed early with the requisite precautions, and a suitable regimen be strictly observed, the gonorrhœal inflammation does not proceed in its usual course, and the disease is cured in two or three days.

---

The Society of Pharmacy, at Paris, has proposed the following Prize Questions on the nature of those preparations termed *Pharmaceutic Extracts;* and, particularly, of the immediate principle in Plants, known by the name of *Extractive Matter*—a principle, the existence of which is as yet only considered as problematical, viz.

1. Does there exist in plants a substance, *sui generis*, different from the immediate principles already known, and which may be termed *extractive?*

2. If this extractive matter does exist, what means have we to isolate it, and what are its characteristic properties?

3. To what substances is it most frequently united in the pharmaceutic extracts? In case the principle termed *extractive* should not exist in plants, it is also desirable that the substances should be stated, the amalgamation of which constitutes the principal extracts.

4. If the extractive matter does exist, what are its relations and differences with respect to the principles forming the colouring matter?

5. What are its operations in chemistry, both with respect to the œconomical and chemical arts in which plants are used?

The answers, signed with a motto only, are directed to be sent, post paid, before the 15th January, 1815, to the General Secretary of of the Society, with a sealed note containing the author's real name and address, and marked with the same motto as the answers to the questions; which note will not be opened unless the answer is found to be entitled to a premium, for which a bequest of 600 francs has been left by the late M. Parmentier, member of the French Institute.

---

M. Montain, physician of the Hotel Dieu, at Lyons, has successfully employed the muriate of ammonia in the treatment of hospital gangrene. Nearly five hundred soldiers, with gun-shot wounds, having been admitted into the hospital, upwards of sixty were affected with this disease. Notwithstanding the use of the usual means, the infection had spread rapidly until this medicine was used. The wound, and more especially the livid parts were first sprinkled with it, and afterwards moistened with camphorated spirits; over this a pledget of lint, with sulphur ointment, or powdered charcoal, was applied, and the whole covered with compresses steeped in camphorated spirits. Various instances of the success of this practice are adverted to.—*Gazette de Santé.*

In the same Gazette is related, by M. Godeman, the case of a young woman in good health, from whom grew *two horns*, which possessed the usual firmness, and were curved from without inwards. The first was situated on the middle of the radius of the right arm, three inches in length, and nearly the same in circumference; the other lower, and not quite so large. They are said to be different from the horny tubercles described by Heliodore Bonn, and in the Philosophical Transactions. The editor adds, that analogous examples are to be met with in Alibert's treatise on diseases of the skin: some are to be found in the Dictionaire des Sciences Medicales, under the title *Cas. Rares.* Conrad Peyer, in a work intitled Merycologia, also relates many instances.

---

*Remedy for Croup.*—The French government having lately offered a large premium for the best essay (memoir) on the nature and successful treatment of the croup; a special committee of Messrs. Dessarts, Portal, Hallé, Pinel, Thouret, Corvisart, Chaussier, Leroux, Duchan, Royer-Collard, and Balleroy, was appointed to examine the papers transmitted. Eighty-three were received, two of which were so highly approved, and considered to possess such equal merit for correctness of pathology and method of cure, that the premium was divided between M. Jurine, Professor of Anatomy and Surgeon, at Geneva, and Dr. John Abraham Albert, of Bremen, Doctor of Medicine and Surgery, and Member of the Imperial and Royal Josephine Academy of Vienna, &c.

The particular doctrines of their important papers are not related, but mention is made of another paper deserving notice, on account of a novel remedy as a specific for this disease, and also for *Pertussis.* The Sulphuret of Potass, commonly called *Hepar Sulphuris,* six grains to be given night and morning, in a little honey or syrup. To be taken as soon as mixed, either by a spoon, or when given to infants, by dipping the finger into it, and permitting them to suck off the medicine. Each dose to be sent in a separate phial, well corked.

 The

The quantity may be increased to ten grains, and gradually diminished with the removal of the complaint. The physicians and surgeons on the committee, however, did not offer any opinion upon this specific so called, but requested to receive further information on its influence both in the croup and catarrhal affection of the fauces; and practitioners in different parts of the country were requested to communicate the result of their observations.

---

*Analysis of the Broad-leaved Tobacco, (Nicotiana Latifolia,) and of the Deadly Night-shade, (Atropa Belladonna,) by* M. VAUQUELIN.—Tobacco contains an animal matter of the nature of albumen, malate of lime, with an excess of acid, acetic acid, nitrate and muriate of potass, a red matter of an unknown nature, muriate of ammonia, and an acid volatile principle different from every other belonging to the vegetable kingdom. This principle gives the tobacco its peculiar characteristic properties, and may be separated from the plant by distillation, and employed for the purposes of tobacco in that state. Prepared tobacco contains more carbonate of ammonia and muriate of lime than the unprepared plant.

As the narcotic properties of the belladonna resemble those of tobacco, Vauquelin supposed it might contain the same acrid principle; but its analysis discovered only an animal matter, some salts with a basis of potass, and a bitter substance on which the narcotic quality of the belladonna depends. In order to ascertain the effects of this substance on the animal economy, Vauquelin gave portions of it to various animals, all of which became intoxicated and delirious, exactly in the same manner as if they had swallowed opium.

---

M. CHEVREUL has examined the bitter principle produced by the action of nitric acid upon organized vegetable substances which contain azote; a subject of investigation which has already engaged the attention of Hausmann, Welther, Proust, Fourcroy, and Vauquelin.

M. Chevreul conceives that this bitter is a compound of nitric acid and an oily or resinous vegetable substance; and he attributes the property which it possesses of detonating to the decomposition of the nitric acid, the formation of ammoniacal gas, prussic acid, and an oily hydrogen gas, &c. &c. an opinion which is partly confirmed by the observations of Fourcroy and Vauquelin. But at the same time that the bitter is formed, a resinous substance and a volatile acid are also produced, upon which M. Chevreul has made many experiments; and which he regards as differing from the bitter ingredient only in containing a smaller proportion of nitric acid.

M. Chevreul has also investigated the nature of the substances formed by the action of nitric acid on carbonaceous or resinous matters, which have the property of precipitating gelatin. The first observations on this subject were made in England by Mr. Hatchett, and the substances obtained were regarded as analogous to tannin. M. Chevreul conceives this opinion to be erroneous, and believes that they differ among themselves not only according to the kind of acid and the substance with which they have been prepared, but also according to the quantity of acid which enters into their composition.

By

By a letter received from Bagdad, dated Aug. 24, 1813, it appears that JOHN MURAT, a native of Constantinople, and a merchant at Bagdad, has had the merit of introducing Vaccination on the Banks of the Tygris and Euphrates. Animated with the desire to be useful to his fellow creatures, in a country where the small-pox, if it be not always mortal, leaves scars and deformities, this humane individual had long wished to communicate the benefits of this discovery to the inhabitants of Bagdad. His endeavours were, however, at first abortive: he had to overcome prejudices, which were armed by a double portion of ignorance in these regions. An accidental circumstance strengthened these prejudices. The first trials of the British resident were unsuccessful, or rather the results were doubtful; these trials were made eleven years ago, and they are deserving of notice.

Seconded by Dr. Short, Mr. Jones, in 1802, obtained some vaccine matter from Lord Elgin and from Dr. Carro. He vaccinated two poor infants, who were procured more by the allurement of money given to their parents, than by any assurances of success to the operation. The infection took in the arm of both; but the English physician not having been sufficiently careful in the choice of these two children, whom distress had placed at his disposal; one, which was healthy, went through the disease regularly; the other, which had been long sickly, died a few days afterwards. This accident having frightened every body, all the efforts of Mr. Jones to procure other children were unavailing, and vaccination was for a time exploded. John Murat's opinions, in the mean time, remained unchanged, and he frequently procured matter from Constantinople, but he did not succeed in getting a single child entrusted to him to vaccinate. At length, in 1810, he had a child born to him, and he determined to convince the inhabitants of Bagdad that vaccination, far from being dangerous, tended to diminish the ravages of small-pox. He sent to Aleppo accordingly for matter, and vaccinated the new-born infant. The experiment answered his expectation, and produced the desired effect upon a great number of Christians, who were witnesses of the operation. Crowds daily attended with their children to be vaccinated. The Turks and Arabs, although contrary to their religious tenets on the score of predestination, speedily followed the example, and ninety-six Turkish and Arab children have been vaccinated in the course of the year (1813). Among other distinguished personages, the Mufti, the Defterdar, and Davoud Effendi, brother-in-law to the pacha of Bagdad, have had their children vaccinated. The pacha has also signified his satisfaction by sending John Murat a handsome present.

Not satisfied with his success at Bagdad, the benevolent Murat has procured missionaries to visit Mesopotamia and Armenia, and has instructed them in the operation, furnished them at the same time with directions printed in Arabic and Armenian, for propagating the benefits of vaccination.

---

The Master, Governors, and Court of Assistants of the Royal College lege of Surgeons, adjudged the Jacksonian Prize for the year 1813, subject "Injuries and Diseases of Nerves," to Mr. Pring, of Wolcot-place, Bath; and decreed an extra premium of equal value to Mr. Henry Earle, of Berner's-street, for his dissertation on the same subject.

---

Dr. SQUIRE will on Tuesday, the 7th of June, begin a Course of Lectures on the Theory and Practice of Midwifery, and the Diseases of Women and Children. Particulars may be known at Dr. SQUIRE's house, Ely Place, Holborn.

---

Dr. CLUTTERBUCK will begin his Summer Course of Lectures on the Theory and Practice of Physic, Materia Medica, and Chemistry, on Wednesday, June 2d, at ten o'clock in the morning, at his house, No. 1, in the Crescent, New Bridge-street, where further particulars may be had.

---

Dr. ADAMS's Summer Course of Lectures will commence on the second Tuesday in June.

---

Dr. HARRISON shortly commences his Summer Course of Lectures on the Clinical cases which occurred in St. George's Hospital; to which will be added the Lectures usually delivered in the schools of medicine. We understand an arrangement has been made, by which the perpetual pupils of Dr. PEARSON's Lectures are entitled to attend Dr. HARRISON, free of expence; and *vice versâ*, the pupils of Dr. H. are entitled to attend the Winter Course of Lectures delivered by Dr. P.

---

*Medical and Chemical Lectures and Examinations.*—Dr. HOOPER and Dr. AGER will begin their next Course on the Theory and Practice of Physic, Chemistry, and the Materia Medica, at the Theatre of Mr. BROOKES, Blenheim-street, Great Marlborough-street, on Monday, June 6th, 1814, at two o'clock. Three Courses are given yearly, beginning in February, June, and October, each on the first Monday of the month.

---

*Theatre of Anatomy, Blenheim-street, Great Marlborough-street.*—Mr. BROOKES will commence his Summer Course of Lectures on Anatomy, Physiology, and Surgery, on Monday the 6th day of June, at seven o'clock in the morning. The Dissecting Rooms will be open at five o'clock in the morning, and continue so until the same hour in the afternoon, attended by Mr. BROOKES.

N. B. The subjects are preserved by an antiseptic process, discovered by Mr. BROOKES, that enables the students to dissect with safety, comfort, and every possible advantage.

The Museum will be open some time during the summer, when every gentleman who wishes to see the collection of preparations, may obtain a ticket of admission by sending his card, expressive of the intention.

The ensuing Summer Course of Lectures on the Theory and Practice of Midwifery, and on the Diseases of Women and Children, by D. D. Davis, M.D. Fellow of the Royal College of Physicians in Edinburgh, Licentiate of the Royal College of Physicians in London, Man-Midwife in Ordinary to the Queen's Lying-in Hospital, &c. &c. will commence on Monday, the 6th of June, at ten o'clock in the forenoon, at Dr. Davis's house, No. 18, Lower Charlotte-street, Bedford-square. A limited number of young gentlemen are admitted as medical house pupils.

